# Mental Health Disorders in Nurses During the COVID-19 Pandemic: Implications and Coping Strategies

**DOI:** 10.3389/fpubh.2021.707358

**Published:** 2021-10-26

**Authors:** Brittney Riedel, Sydney R. Horen, Allie Reynolds, Alireza Hamidian Jahromi

**Affiliations:** ^1^Department of Nursing, Northwestern Memorial Hospital, Chicago, IL, United States; ^2^Division of Plastic and Reconstructive Surgery, Rush University Medical Center (RUMC), Chicago, IL, United States; ^3^Princeton University, Princeton, NJ, United States

**Keywords:** mental health disorders, COVID-19, pandemic, coping skills, health care workers, health care providers

## Abstract

Nurses caring for patients who contract coronavirus disease 2019 (COVID-19) have experienced significant traumas in the form of increased workloads, negative patient outcomes, and less social support system access. Nurses should be provided with information regarding early detection, coping skills and treatment for anxiety, depression, post-traumatic stress syndrome (PTSS)/post-traumatic stress disorder (PTSD), and other mental health disorders. Early intervention is important as mental health disorders can cause dysfunction, internal suffering, and in the most extreme situations, lead to death if not properly cared for. Healthcare corporations should consider providing coverage for mental health treatment for employees who experience COVID-19 traumas. With the implementation of healthy coping skills and therapeutic intervention, nurses will be able to let go of the negative impacts that the COVID-19 pandemic has caused and reintegrate into their roles as caring and entrusted health care providers. The current paper evaluates the mental health disorders encountered by nurses in the COVID-19 era based on the current medical literature and aims to provide practical coping strategies.

## Introduction

According to the Gallup's Most Honest and Ethical Professions Poll, for the last 19 years, Americans have rated nurses highly regarding honesty and ethical standards (1). Nurses are held to a high ethical standard and entrusted with a significant amount of patient care duties. During the Coronavirus 2019 (COVID-19) pandemic, expectations have risen, with greater emphasis has been placed on public health decision-making. Health care providers (HCP), including nurses, have been under a tremendous amount of pressure and have continued to be the society leaders in guiding the public during the pandemic. Nurses have been asked to pick up extra shifts, work late, and skip breaks all in a continuously high-stress environment. The patient-to-staff ratio is strained considering the number of medically unstable COVID-19 patients, and nurses have experienced negative patient outcomes and death at high rates. Under normal situations, nurses devote extra time to patients who are severely ill or those who are psychologically struggling with their illness. However, during the COVID-19 pandemic, this has not been possible. Nurses have not been able to provide their usual standard of care or emotional support which goes against their core nature; to help others to the best of their capabilities. Nurses have also been working at a faster pace due to the high burden of COVID-19 admissions. When there is a negative outcome, the COVID-19 unit often does not have time to debrief, which does not allow time for individuals to fully process the trauma, experience grief, and subsequently recover (2, 3).

These conditions can impact the mental health of nurses and lead to the development of depression, anxiety, post-traumatic stress symptoms (PTSS), post-traumatic stress disorder (PTSD), and other mental health disorders. Prior to COVID-19, nurses may have been visited by family and friends, routinely exercised outdoors or in the gym or been involved in social group activities such as athletic teams or extracurricular clubs. However, social distancing has limited typical coping skills used for handling difficult, stressful, traumatic, and emotionally exhausting situations. These traumatic events often have a delayed impact on an individual's mental health. Those who have been traumatized by the COVID-19 pandemic may not currently be aware of the negative implications they will face in the future. Mental health awareness is critical as trauma will often impact work performance. Individuals may experience, irritability, sleep changes, and social or communicative withdrawal after traumatic events (4).

Working under poor conditions while not being able to provide excellent care to patients has been and will continue to impact the mental well-being of nurses as well as other HCP. The novel and overwhelming aspects of added ethical and patient care responsibility has contributed to an increased development of mental health disorders in nurses (5). The consequences of overlooking this problem could be devastating. Nurses must be supported to sustain a healthy mindset during these unprecedented times (5). This may be done through increased awareness of coping skills and professional services which can provide mental health relief related to the negative impacts of COVID-19. The current paper evaluates the mental health disorders encountered by the nurses during the COVID-19 era based on the current medical literature, assesses its implications, and aims to provide practical coping strategies.

## Mental Health Disorders During COVID-19 and Past Pandemics

To help nurses cope with COVID-19 related trauma, the relationship between COVID-19 and the development of PTSS and PTSD must be elucidated. Studies have shown that during the outbreaks of severe acute respiratory syndrome (SARS), Middle East respiratory syndrome Coronavirus (MERS-CoV), and COVID-19 the development of PTSD-like symptoms have ranged between 11 and 73.4% with 51.5% of HCPs scoring above the Event Scale-Revised (IES-R) threshold for PTSD diagnosis. It was also found that HCPs during the COVID-19 pandemic have had a higher rate of PTSD-like symptoms (71.5–73%) when compared to rates during the SARS outbreak (5%) (6).

One study predicted that between 10 and 40% of HCP will have a manifestation of PTSD between 1 and 3 years after a pandemic (6). This rate is particularly concerning when considering that coping skills and treatments for PTSD/PTSS are not easily assessable or well-known within the HCP community.

A recent study by Chen et al. investigated the development of PTSD-like symptoms in nurses working with COVID-19 patients. It was found that women nurses, working in ICUs and COVID-19 designated hospital and departments were at the highest risk of developing PTSD-like symptoms (7). In a meta-analysis looking at the prevalence of anxiety, depression, and insomnia during COVID-19, rates were found to be 23, 28, and 39%, respectively (8). A higher rate of affective symptoms was reported by nurses and female HCP. These findings suggest that hospitals should prioritize appropriate psychological support and treatment for these specific at-risk HCP groups.

PTSD and PTSS are not the only mental health disorders that HCP may experience related to COVID-19. A study by Zhang et al. in 2020 showed that a large percentage of HCP who developed PTSD during the COVID-19 pandemic also experienced higher rates of anxiety (30.71%) and depression (71.26%) (4). Anxiety and depression can have lasting and devastating consequences. Krishnamoorthy et al. (9) published a systematic review and meta-analysis looking at the mental health of HCP and found that stress, psychological distress, sleep quality, insomnia, anxiety, and depression were all increased secondary to COVID-19.

Aside from HCP, patients who had suffered from a COVID-19 infection were the only group to have a greater psychological burden reported. Furthermore, nurses and other HCP may be more likely to be exposed to COVID-19 due to their close proximity with sick patients. In a study looking at HCP who were exposed to SARS, MERS, or COVID-19, Salazar et al. (10) found that psychological distress (38%), fear (44%), anxiety (29%), depression (26%), PTSD (21%), somatization (16%), and burnout (34%) were all increased. Poor working conditions and the possibility of a COVID-19 exposure have a clear negative impact on the mental health of HCP.

## Risk Factors for the Development of Mental Health Disorders in HCP

The United States (US), when compared to 16 other countries, has one of the highest reported rates of exposure to traumatic events, defined as the “exposure to actual/threatened death, serious injury or sexual violation leading to flash backs, avoidance, negative cognitions, mood, and arousal symptoms” (11). While not all individuals who encounter trauma go on to develop PTSD, the populations most at risk for developing this disorder are younger females, those lacking social support, and economically marginalized individuals (11). Since a large proportion of the nursing population consists of young female workers who lack social support due to social distancing restrictions, this population is at a higher risk of developing PTSD in the current COVID-19 crisis (11).

In addition to national social distancing protocols implemented during the COVID-19 pandemic, nurses may be even further impacted due to self-imposed isolation practices. Some nurses have been living away from home in hotels so as to not put their family members at risk of contracting COVID-19. This extreme degree of isolation may contribute to an even greater percentage of nurses being affected by mental health disorders (12).

In a study by Cui et al., the impact of socio-psychological and working condition variables on the development of anxiety in nurses during COVID-19 was evaluated. It was found that nurses who were female, had less rest time, and lacked confidence in fighting the pandemic were at risk for developing anxiety. In addition, while nurses with families at home may experience greater levels of social support, they were also more likely to develop anxiety as they feared infecting these family members after working on the COVID-19 front-line (13).

**Figure 1:**
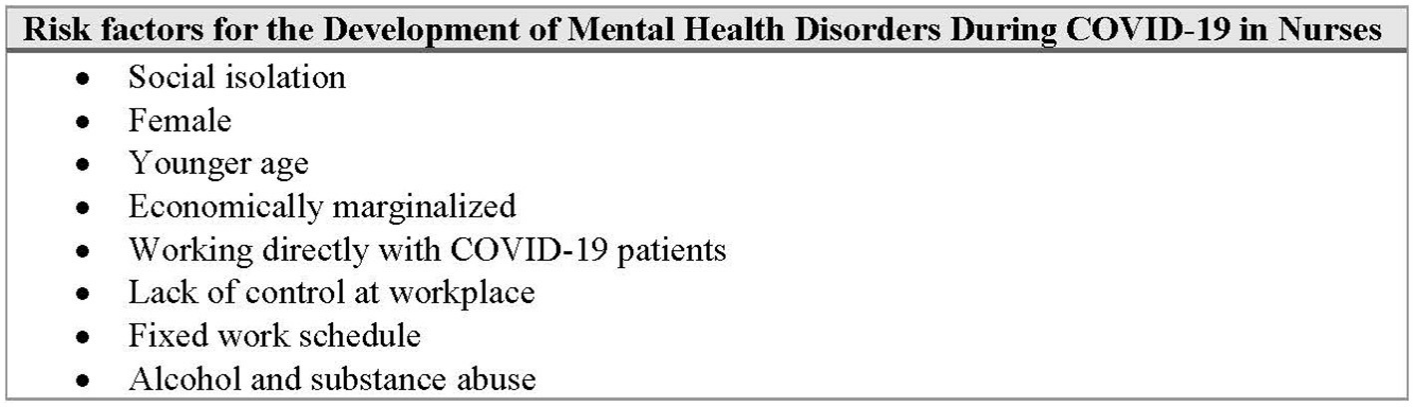
Risk factors for the development of mental health disorders during COVID-19 in nurses.

Saeed et al. investigated risk factors associated with depression in South Asian HCP during COVID-19 and found that female gender, younger age, and a fixed working schedule were independent predictors of depression. Female HCP were 1.6 times more likely to develop depression compared to their male colleagues, hypothesized to be due to the difficult balance of home and work responsibilities. In young HCPs, depression was more common potentially due to more difficult and demanding work duties as compared to senior colleagues. It was also found that nursing staff specifically was most at risk for the development of depression. Given that nurses are often, young, female, and have fixed work schedules, this study mirrors the previous findings (14) (Figure 1).

## Support During the COVID-19 Pandemic

A weak social support system is one of the greatest risk factors associated with the development of PTSS/PTSD (4). During the SARS outbreak, social and familial support was found to be protective against the development of PTSS/PTSD and nurses who had a strong support system were less likely to develop acute stress disorders which positively impacted their daily activities (4). Individuals who had someone to talk to regarding their work experiences were less at risk of developing PTSS, while individuals who tended not to vent about their experiences had higher levels of PTSS (15, 16). During the South Korea MERS outbreak, it was found that supervisor and colleague support was beneficial in the reduction of PTSS (15, 16).

In a systematic review by Sirois et al. (17) in which factors associated with psychological distress in HCP during an infectious disease outbreak were examined, 19 studies that specifically looked at the impact of social support found that this was a key protective factor. A meta-analysis revealed decreased social support was one of the most important risk factors among the 25 potential risk factors of PTSD. Having access to adequate economic assistance, psychological interventions, and sufficient social support may help alleviate PTSD symptoms in HCP (4). Similar findings were observed during the SARS outbreak as a greater level of family support was associated with the reduction of depression and anxiety levels (18). During the MERS outbreak poor family/friend support was associated with higher levels of burnout (19). During pandemics, having a strong social support system helped protect nurses against the development of acute stress symptoms (15, 16). Whether support was from family, friends, supervisors, and colleagues, it significantly decreased mental health disorders. In studies during the COVID-19 pandemic, social support significantly lowered levels of PTSD, anxiety, depression, and stress (20, 21).

In a study by Heath et al., social support before and during traumatic events was seen to decrease psychological injury experienced by HCP. It was also found that HCPs who had meaningful personal and professional relationships had a lower risk of burnout (22). As burnout is often related to increasing levels of stress, anxiety, and depression, social support may substantially decrease the burden of these mental health disorders leading to greater job satisfaction and job retention.

Organizational support in addition to personal, familial, or friend relationships has also been shown to buffer the development of mental health disorders. In nurses working during the SARS pandemic in Canada, the perception of a high level of organization support (i.e., positive feedback on job performance given by co-workers or doctors) was correlated with decreased levels of emotional exhaustion and overall positive attitudes toward the outcomes of the virus (23). During the SARS, MERS and COVID-19 pandemic, HCP who had positive perceptions toward colleagues and supervisors had lower levels of psychiatric symptoms such as PTSD and distress (17).

## Methods

A review of the medical literature was performed to find articles proposing strategies to cope with mental health related issues that may occur in the context of a pandemic. Due to the extensive nature of this topic with a wide range of disorders and strategies, a systematic review was not performed. However, the medical literature was extensively analyzed so as to provide an overview of the following coping strategies: mindfulness and moral resilience, cognitive behavioral therapy, cognitive processing therapy, emotional freedom technique, prolonged exposure and eye movement desensitization and reprocessing therapy, and motor interference therapy and traumatic memories. The authors searched for these terms and selected articles that were applicable to the mental health disorders experienced in the context of the COVID-19 pandemic and were practical in implementation. In addition to an extensive literature search, the references of those publications that were reviewed were also reviewed so as to capture all relevant articles. Overview of these common coping strategies aimed to allow for better resilience and fortitude for healthcare workers suffering from COVID-19 related mental health disorders.

## Coping Strategies for Mental Health Disorders

### Mindfulness and Moral Resilience

**Figure 2:**
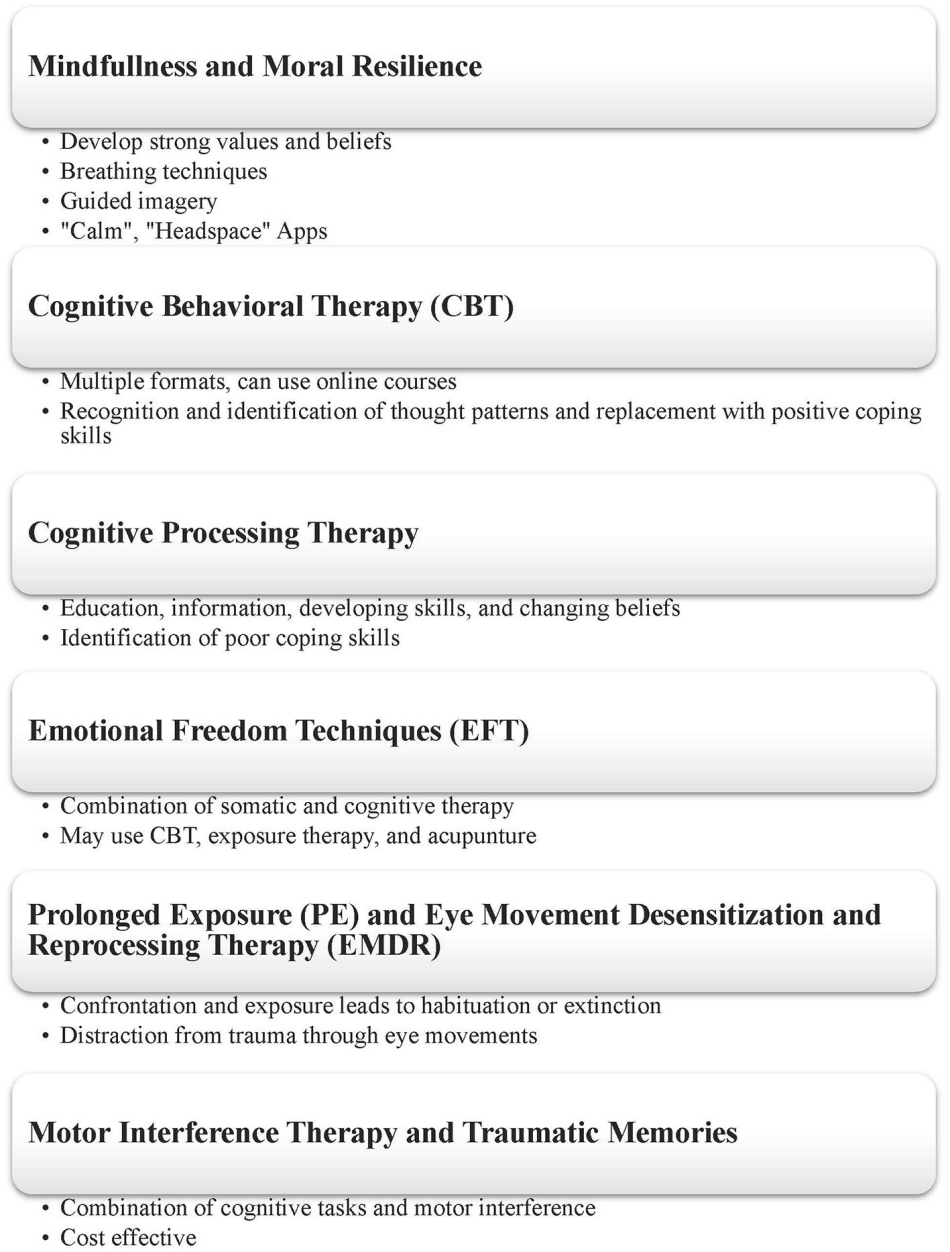
Overview of coping strategies used to prevent the development of mental health disorders during COVID-19.

Nurses should focus on developing moral resilience when caring for patients with COVID-19. Moral resilience describes when one is able to confront distressful and uncertain situations with courage and confidence through reliance on a strong system of values and beliefs (Figure 2). Moral resilience helps keep individuals in check, allowing the mind to contextualize a situation and understand when events are out of one's control. The concept of moral resilience must be gradually built and developed by an individual and requires persistence and experience. Strategies that help build moral resilience include practicing mindfulness and focusing on strengthening the parasympathetic nervous system to positively react to stress (i.e., through deep breathing exercises) (5).

Mindfulness is a form of meditation where an individual focuses on current sensations and feelings in a specific moment without applying any thoughts to those feelings. While focusing on one's senses and feelings, the individual can apply relaxing practices such as breathing techniques and guided imagery to help the body and mind remain calm. This results in mental clarity and reduction of stress. In conjunction with moral resilience, practicing mindfulness has been shown to help calm the sympathetic nervous system by lowering stress hormones such as epinephrine, norepinephrine, and cortisol which are involved in the body's fight-or-flight response (24).

Focused breathing excises and keeping a clear and calm mindset prior to entering the room of a patient can help nurses tolerate inevitable stress and trauma that they will face. With nurses focused on work instead of anxiety, stress, and fear they can provide better patient care with greater efficiency. The development of mindfulness and parasympathetic nervous system strengthening strategies can be achieved through apps such as “Calm” or “Headspace.” With the help of these tools, nurses can respond to ethical issues in a more positive way (5).

### Cognitive Behavioral Therapy (CBT)

Cognitive Behavioral Therapy (CBT) has also been shown to be effective in the treatment of depression, anxiety, and PTSD. This therapy is a collaborative psychological treatment that may be given in various formats (group, individually) and with various levels of support systems present (i.e., parents, family, friends). CBT intervention involves the recognition of certain emotions or feelings, the identification of thoughts or triggers associated with the feeling, and the development of coping skills to use when the feelings are experienced. Coping skills may include modifying negative self-talk into positive-self talk, problem solving skills, and relaxation training, among others. In a systematic review and meta-analysis on the use of CBT in individuals with anxiety, it was found that CBT increases post-treatment remission in all anxiety diagnoses (25). It was also found that for patients with anxiety specifically, CBT was found to potentially be more effective than other attention control therapies (25).

In a study looking at the effects of CBT on sleep quality in HCP during the COVID-19 pandemic, it was found that an online CBT course improved sleep quality (26). As poor sleep quality often can contribute to mood disorders, and vice-versa, using CBT to improve this function may have benefits for HCP experiencing the early or more advanced symptoms of a mental health disorder. In a similar study by Weiner et al., the authors performed a randomized controlled trial to investigate the impact of online CBT on the stress levels, depression, and insomnia in HCP. It was found that a brief CBT program significantly decreased perceived stress levels and improved or prevented severe psychiatric disorders like PTSD and depression (27). The user friendly and easily accessible online CBT platforms that these studies mention may allow for nurses and other HCP to use CBT while still safely practicing social distancing. HCPs may also access CBT at convenient times that fit into their busy schedules.

### Cognitive Processing Therapy

For individuals who have experienced trauma, cognitive processing therapy may be beneficial. Cognitive processing therapy is a form of CBT that follows a four-step process: education, information, developing skills, and changing beliefs. Using this approach in conjunction with a trauma therapist, nurses can start to identify mental health disorder symptoms and better understand how to recognize their thought and feeling patterns. Cognitive processing therapy can also help nurses understand how past traumas are directly linked to the stress, anxiety, and depression they are experiencing (5).

Individuals may greatly benefit from being self-aware of poor coping skills and addressing them appropriately. One study found that nurses and doctors started consuming alcoholic beverages at higher consumption rates and with greater frequency during the COVID-19 pandemic (28). Nurses should be extra vigilant regarding substance abuse as the profession is often ranked among the highest out of all professions for the prevalence of substance abuse (29). Developing poor coping skills during the COVID-19 pandemic could result in negative long-lasting and possibly fatal consequences for individuals. Nurses should be encouraged to spot poor coping skills and seek proper support and treatment accordingly. Cognitive processing therapy is among the many strategies that may be used to identify and alter negative habits. Through education and gathering information regarding the negative impacts of certain habits and developing skills to counteract them, nurses can change their beliefs, and develop more positive ways to deal with trauma.

### Emotional Freedom Techniques (EFT)

Another common therapy found to be effective for the treatment of PTSD/PTSS is emotional freedom techniques (EFT). This therapy combines somatic and cognitive therapy in the form of CBT, exposure therapy, and in some cases a form of acupuncture. In a past study looking at the psychological impact of EFT on veterans with PTSD, outcomes were excellent with a significant reduction in psychological distress and PTSD symptoms reported (30). In the same study, when patients who received EFT were compared with those who did not, 90% of the EFT group vs. 4% of the non-intervention group did not meet PTSD clinical criteria. Educating nurses about the efficacy of EFT and providing access to these therapies and techniques ensures that mental health is being considered with a high priority (5).

### Prolonged Exposure (PE) and Eye Movement Desensitization and Reprocessing Therapy (EMDR)

Two popular evidence-based trauma-focused treatments used for individuals suffering from PTSD are prolonged exposure therapy (PE) and eye movement desensitization and reprocessing therapy (EMDR). PE therapy consists of a patient being instructed by a therapist to confront traumatic memories and expose themselves continuously to fearful stimuli with the goal of reaching habituation or extinction (31).

In EMDR therapy, patients are distracted from their disturbing or traumatic memories by using a dual attention task which typically involves eye movements. As these therapies target trauma in different way, individuals suffering from PTSD often benefit from implementing both PE and EMDR in a dual treatment setting. One study indicated individuals who participated in twice a day therapy sessions, with a PE session in the morning and a EMDR in the evening, were found to have higher satisfaction rates and reduced PTSD symptoms (31). The study concluded that PE tends to activate higher rates of fear in PTSD individuals whereas EMDR has been shown to reduce fear and leave a patient feeling relieved and satisfied. Providing PE sessions first and ending the day with EMDR helps an individual's overall levels of fear dissipate throughout the day so that their mind is relaxed at night and ready for sleep (31).

HCPs may benefit from combining PE and EMDR as a treatment option for the PTSS/PTSD symptoms related to the COVID-19 pandemic. One major benefit of both PE and EMDR is that treatment sessions are relatively short-term and shown to be highly effective. In previously mentioned study, patients underwent combined therapy for 2 weeks with a total of eight treatment days. Results showed that PE and EMDR were highly effective in reducing the severity of PTSD symptoms (*r* = 0.59, *p* < 0.001) (31). Having a relatively short-term treatment is convenient for nurses who are often extremely busy with their personal and professional lives. This is also unique as other standard cognitive therapies such as talk therapy may be conducted over the course of months or even years.

EMDR can be used to help reduce anxiety and depression associated the PTSD (11). A review on the topic found that EMDR was more successful at treating anxiety linked to PTSD than CBT. Individuals who are more comfortable with the traditional CBT approach should consider this option for coping with the negative effects of the COVID-19 pandemic, however, supplementing with EMDR therapy may further improve symptoms.

### Motor Interference Therapy and Traumatic Memories

Motor inference therapy is effective in treating patients suffering from past traumas. This therapy is inexpensive and involves the combination of cognitive tasks and motor interference (such as finger-tapping). This therapy is similar to EMDR in that a certain task can be used to distract a patient and positively interfere with their traumatic memory processing. One experiment had patients with PTSD listen to audio stimuli which instructed them to finger-tap to certain sounds/cues while recalling their traumatic event. At 1-week post-treatment, 30% of individuals no longer met criteria to be diagnosed with PTSD (32). Like EMDR and PE, motor interference therapy only requires a short duration of treatment and is cost-effective. Therefore, this therapy may be an ideal treatment option for nurses who experience PTSD/PTSS from COVID-19.

## Discussion

Nurses caring for patients who contract COVID-19 have experienced significant traumas in the form of increased workloads, negative patient outcomes, and less social support system access. Mental health awareness should be discussed in both the workforce and personal social settings. Nurses should be provided with information regarding coping skills and treatment for anxiety, depression, PTSS/PTSD, and other mental health disorders. Nurses should be aware of the preliminary signs and symptoms of mental illnesses. Early intervention is important as mental health disorders can cause dysfunction, internal suffering, and in the most extreme situations, lead to death if not properly cared for. The COVID-19 pandemic has isolated healthcare workers in ways that are challenging for the general public to comprehend. Healthcare corporations should consider providing coverage for mental health treatment for employees who experience COVID-19 traumas. Institutions should reiterate to nurses and other HCP that they are not alone, there is hope, and that mental health will improve with the seeking of help and time. Due to isolation and social distancing, it is not uncommon to feel alone and hopeless during the COVID-19 pandemic. However, as a community we must support our HCP and make sure they have access and support for whatever care they may need. With the implementation of healthy coping skills and therapeutic intervention nurses will soon be able to let go of the negative impacts the COVID-19 pandemic has caused and reintegrate into their roles as caring and entrusted HCP.

## Author Contributions

BR and AH conceptualized the paper and wrote the first editions of the manuscript. All authors contributed to the manuscript with their expertise, also read and edited the submitted version, and approved the submitted version.

## Conflict of Interest

The authors declare that the research was conducted in the absence of any commercial or financial relationships that could be construed as a potential conflict of interest.

## Publisher's Note

All claims expressed in this article are solely those of the authors and do not necessarily represent those of their affiliated organizations, or those of the publisher, the editors and the reviewers. Any product that may be evaluated in this article, or claim that may be made by its manufacturer, is not guaranteed or endorsed by the publisher.
